# Cholesterol metabolic reprogramming drives the onset of DLBCL and represents a promising therapeutic target

**DOI:** 10.3389/fcell.2025.1585521

**Published:** 2025-09-17

**Authors:** Lili Zhou, Wei Cheng, Dan Luo, Zhida Peng, Jiaqi Mei, Qingqing Luo, Tiantian Yu, Ya Wang, Zhixiang Lei, Chunhong Huang, Nianlong Yan, Daya Luo, Li Yu

**Affiliations:** ^1^ Department of Hematology, The Second Affiliated Hospital, Jiangxi Medical College, Nanchang University, Jiangxi Provincial Key Laboratory of Hematological Diseases (2024SSY06052), Nanchang, Jiangxi, China; ^2^ Department of Biochemistry and Molecular Biology, School of Basic Medical Sciences, Jiangxi Medical College, Nanchang University, Nanchang, Jiangxi, China; ^3^ Department of Hematology, Jishou University First Affiliated Hospital, Jishou, China; ^4^ Department of Pathology, Eye Hospital affiliated with Nanchang University, Nanchang, China

**Keywords:** diffuse large B-cell lymphoma, cholesterol metabolism, prognosis, survival, serumlipids

## Abstract

**Background:**

Cholesterol is an essential molecule for tumor cell growth and proliferation, and dysregulated cholesterol metabolism has been widely implicated in cancer pathogenesis. However, the specific role and underlying molecular mechanisms of cholesterol metabolism alterations in diffuse large B-cell lymphoma (DLBCL) remain poorly understood.

**Methods:**

We retrospectively analyzed clinical data from 200 DLBCL patients and 185 healthy controls, focusing on lipid and lipoprotein levels, including triglycerides (TG), total cholesterol (TC), low-density lipoprotein cholesterol (LDL-C), high-density lipoprotein cholesterol (HDL-C), apolipoprotein A1 (ApoA1), apolipoprotein B (ApoB), and apolipoprotein E (ApoE). Univariate and multivariate Cox proportional hazard models were used to evaluate the prognostic value of these markers, and Kaplan-Meier analysis assessed their associations with overall survival (OS). Bioinformatics analysis predicted associations between lipid markers and cholesterol metabolism. Cellular experiments further investigated the expression of cholesterol metabolism-related proteins and the effect of the cholesterol-depleting agent Methyl-β-cyclodextrin (MβCD) on DLBCL cells.

**Results:**

We confirmed significant alterations in metabolic markers (such as TC and ApoA1) between the healthy control group and patients, which were significantly associated with patient prognosis and overall OS. Bioinformatics analysis revealed a strong correlation between these markers and elevated CD36 expression. In addition, DLBCL cells exhibited increased expression of cholesterol uptake and synthesis proteins (CD36, SREBP2, and HMGCR) and decreased expression of efflux proteins (APOA1, NR1H2 and ABCG1), consistent with cholesterol metabolic reprogramming. Treatment with MβCD disrupted CD36 expression and cholesterol metabolism, leading to reduced DLBCL cell survival.

**Conclusion:**

These findings underscore the pivotal role of cholesterol metabolic reprogramming in DLBCL progression. CD36 and related metabolic markers represent promising therapeutic targets, opening novel avenues for the treatment of this malignancy.

## 1 Introduction

Diffuse large B-cell lymphoma (DLBCL) is the most common type of non-Hodgkin lymphoma (NHL) in adults, characterized by significant clinical and prognostic heterogeneity. Despite advances in treatments such as immunotherapy, CAR-T therapy, and transplantation, which have improved outcomes for many patients, approximately 30%–40% of DLBCL cases still show treatment resistance or relapse (Klener and Klanova, 2020). Current prognostic markers, including chromosomal aberrations and gene mutations, have been pivotal in guiding therapeutic decisions (Reddy et al., 2017; Lenz and Staudt, 2010). However, further enhancement of patient survival outcomes requires the identification of additional factors influencing DLBCL treatment and prognosis. Emergingevidence highlights the critical role of metabolic reprogramming, particularly alterations in cholesterol metabolism and cancer progression. Cholesterol, an essential molecule for maintaining cell membrane integrity and signaling, has been widely implicated in the pathogenesis of various cancers (Huang et al., 2020). Dysregulation of cholesterol metabolism supports tumor cell growth and proliferation and promotes immune evasion by altering the tumor microenvironment (Snaebjornsson et al., 2020; Ma et al., 2019).

However, the mechanisms by which cholesterol metabolism influences DLBCL remain poorly understood. Previous studies have shown that high-density lipoprotein cholesterol (HDL-C) levels are inversely associated with the risk of non-Hodgkin lymphoma (Lim et al., 2007), whereas low serum apolipoprotein A1 (APOA1) levels are significantly correlated with poor prognosis in DLBCL patients (Huang et al., 2024). These findings suggest that cholesterol metabolism could serve as a potential therapeutic target for DLBCL. Nevertheless, the specific role of cholesterol metabolism in DLBCL pathogenesis and progression requires further investigation.

In this study, a retrospective analysis of clinical parameters from 200 DLBCL patients and 185 healthy controls was conducted to systematically investigate the correlation between cholesterol metabolism and DLBCL prognosis. The focus was on evaluating changes in serum lipid levels before and after treatment, including triglycerides (TG), total cholesterol (TC), HDL-C, low-density lipoprotein cholesterol (LDL-C), lipoprotein A (Lp(a)), ApoA1, apolipoprotein B (ApoB) and apolipoprotein E (ApoE). By integrating bioinformatics analysis with *in vitro* experiments, it was found that alterations in lipid metabolites are closely associated with cholesterol metabolic reprogramming, a key feature of DLBCL. Lipid metabolic markers significantly impact patient prognosis. Furthermore, CD36 and cholesterol metabolism-related markers were identified as potential therapeutic targets, providing new insights for DLBCL treatment.

This study combines clinical cases analysis with bioinformatics and fundamental research to elucidate the role of cholesterol metabolism in DLBCL pathogenesis and explore the therapeutic potential of targeting cholesterol metabolic pathways in this malignancy.

## 2 Methods

### 2.1 Patients

According to the diagnostic criteria established by the World Health Organization (WHO), 200 patients diagnosed with DLBCL at the Second Affiliated Hospital of Nanchang University between October 2010 and December 2023 (112 males and 88 females; median age, 61 years) were included in the DLBCL group for retrospective analysis. Additionally, 185 age- and gender-matched healthy individuals were recruited from the health examination center of the same hospital to serve as the control group. The demographic characteristics of the patients are provided in Table 1. All data in this study were obtained in compliance with the ethical principles of the Declaration of Helsinki and approved by the Institutional Ethics Committee of the Second Affiliated Hospital of Nanchang University, Jiangxi, China.

**TABLE 1:** The clinical characteristics of patients with DLBCL.

Variable	N (%)/M (25%, 75%)
Male	112 (56.00%)
Age (y)	61 (52, 70)
IPI (>2)	70 (35.00%)
ECOG (≥2)	30 (15.00%)
Non-GCB	123 (61.50%)
Ann Arbor stage (≥III)	117 (58.50%)
Statins (“yes”)	38 (19.00%)
Diabetes (“yes”)	53 (26.50%)
WBC (×10^9^/L)	6.04 (4.82, 7.44)
Neut (×10^9^/L)	3.78 (2.92, 4.98)
Lym (×10^9^/L)	1.20 (0.82, 1.69)
RBC (×10^12^/L)	4.26 (3.77, 4.68)
Hb (g/L)	125.50 (110.00, 138.75)
PLT (×10^9^/L)	210.00 (164.25, 267.00)
LDH (U/L)	202.46 (164.89, 353.28)
TG (mmol/L)	1.43 (1.03, 2.00)
TC (mmol/L)	4.23 (3.56, 4.94)
HDL-C (mmol/L)	1.01 (0.83, 1.31)
LDL-C (mmol/L)	2.49 (2.14, 3.06)
Lp (a) (mg/dL)	17.64 (8.47, 32.61)
APOA1 (g/L)	1.03 (0.84, 1.27)
APOB (g/L)	0.86 (0.70, 1.03)
APOE (mg/L)	41.29 (32.63, 51.72)

The inclusion criteria for DLBCL patients in this study were as follows: 1) cases diagnosed as DLBCL based on the 2016 WHO criteria (Zhou et al., 2018); 2) newly diagnosed DLBCL patients who had not previously received any anticancer treatment; 3) patients who received at least one cycle of standard treatment after admission; and 4) availability of complete treatment and follow-up records. The exclusion criterion was as follow: presence of other malignancies.

A *post hoc* power analysis was performed using Schoenfeld’s formula, which estimated a statistical power of 79% to detect a hazard ratio (HR) of 0.40, given 48 events, a prevalence of 25.5% in the low-cholesterol group, and a two-sided α level of 0.05.

### 2.2 Data acquisition and processing

In this study, cholesterol metabolism-related genes were identified through a literature review and validated using KEGG enrichment analysis via the website http://sangerbox.com/home.html. Data from 87 healthy controls and 76 DLBCL patients’ blood samples from the GSE83632 dataset in the Gene Expression Omnibus (GEO) database (https://www.ncbi.nlm.nih.gov/geo/) were extracted using www.aclbi.com, and a heatmap of cholesterol metabolism gene expression was created using the “pheatmap” package in R. Data from 223 DLBCL patients in the GSE83371 dataset were also retrieved from the GEO database, and the optimal cutoff value for CD36 was determined using X-tile software (Yale University, CT, United States). The prognostic efficacy of CD36 was evaluated through Kaplan-Meier analysis with a two-sided statistical test. Immunohistochemical results of CD36 in three normal lymph nodes and three cases of NHL were obtained from https://www.proteinatlas.org/.

### 2.3 Cell lines and culture

In this study, the normal lymphocyte cell line GM12878 and DLBCL cell lines WSU-DLCL-4, OCI-LY3, SU-DHL-4, and DB were purchased from Cellverse Co., Ltd (Shanghai, China). Of these, GM12878, WSU-DLCL-4, SU-DHL-4, and DB cell lines were cultured in RPMI 1640 complete medium, whereas the OCI-LY3 cell line was cultured in IMDM complete medium. All cells were maintained in a humidified incubator at 5% CO_2_. Cell line identity was confirmed through short tandem repeat (STR) profiling, and tests were performed to ensure the absence of fungal and *mycoplasma* contamination.

### 2.4 Cell viability assay

To assess cell viability, cells were seeded into 96-well plates and treated with the specified drugs for 24, 48, or 72 h. Subsequently, Cell Counting Kit-8 (CCK-8; Dojindo, Kumamoto, Japan) was added, and absorbance was measured at 450 nm using a microplate reader (Thermo Fisher Scientific, United States).

### 2.5 RT-PCR

Total cellular RNA was extracted using the TRIzol™ reagent (Takara, Japan). RNA from cells treated with MβCD was harvested 48 h post-treatment. Reverse transcription was performed using a commercial kit (AE311-02, TransGen Biotech, China). For RT-PCR analysis, the synthesized cDNA served as template, and PCR amplification was carried out using 2×Taq PCR MasterMix (G3326-15, ServiceBio, Wuhan, China).

### 2.6 Western blotting

Cells were seeded in culture flasks and treated with the specified drugs for 48 h before collection. Total proteins were extracted using cell lysis buffer (Sigma-Aldrich, St. Louis, MO, United States) and quantified using a bicinchoninic acid (BCA) assay kit (ServiceBio, China). Equal amounts of proteins were separated by sodium dodecyl sulfate-polyacrylamide gel electrophoresis (SDS-PAGE) and transferred to polyvinylidene fluoride (PVDF) membranes (Millipore, Billerica, MA, United States). The PVDF membranes were blocked with 5% (0.05 g/mL) skim milk and incubated with primary antibodies (Supplementary Table S1) at 4°C overnight. After washing, the membranes were incubated with secondary antibodies (Supplementary Table S1) for 60 min, and signals were detected using an ECL luminescence reagent (ShareBio, China).

### 2.7 Transfection

The siRNA sequences targeting CD36 were designed and synthesized by Tsingke Biotechnology Co., Ltd. (China). The sequences are as follows: siCD36-1: Sense: 5′-CGACAUGAUUAAUGGUACAdTdT-3′; Antisense: 3′-dTdTGCUGUACUAAUUACCAUGU-5′. siCD36-2: Sense: 5′-GGACCAUUGGUGAUGAGAAdTdT-3′; Antisense: 3′-dTdTCCUGGUAACCACUACUCUU-5′. The transfection of siRNA was performed using the Suspended Cell Nucleic Acid Transfection Reagent (#21032, BIOG, China). RNA was extracted at 24 h post-transfection, and protein was harvested at 48 h for subsequent validation.

### 2.8 Statistical analysis

In this study, all statistical analyses were conducted using IBM SPSS version 25.0 (IBM Corporation, Armonk, NY, United States), GraphPad Prism version 9.5.1 (GraphPad Software, CA, United States), and the R programming language (R Core Team, Vienna, Austria). Comparisons of categorical variables were made using the chi-square test, while continuous variables were analyzed using the t-test (for normal distribution) or non-parametric tests (for non-normal distribution) based on data distribution. After stratifying eight lipid indicators using X-tile software (Yale University, CT, United States), prognostic efficacy was evaluated using the Kaplan-Meier analysis with a two-sided statistical test. Additionally, univariate and multivariate Cox regression analyses were performed to determine the statistical relationships between independent variables and survival rates, with multivariate analysis incorporating variables showing significance in univariate analysis. The Cox proportional hazards regression model was used to assess the impact of lipids as independent risk factors, expressed as hazard ratios (HR) with 95% confidence intervals (CI).

## 3 Results

### 3.1 The difference of serum lipid between DLBCL patients and healthy controls

**FIGURE 1:**
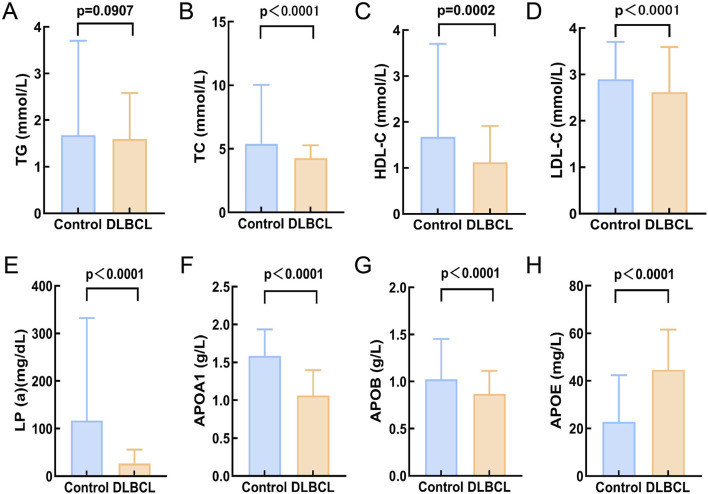
Comparison of serum lipid levels between DLBCLpatients and healthy controls. **(A)** There was no significant difference in TG levels between helthy control and DLBCL (p > 0.05); **(B-G)**:The expression levels of TC, HDL-C, LDL-C, Lp(a), APOA1, and APOB in DLBCL were significantly lower compared to the healthy control group (p < 0.05); **(H)**The expression level of APOE in the DLBCL was significantly higher than that in healthy control group (p > 0.05). Abbreviations: TG, Triglyceride; TC, Total Cholesterol; HDL-C, High Density Lipoprotein Cholesterol; LDL-C, Low Density Lipoprotein Cholesterol; Lp(a), Lipoprotein A; APOA1, Apolipoprotein A1; APOB, Apolipoprotein B; APOE, Apolipoprotein E.

Based on the inclusion and exclusion criteria, a total of 200 DLBCL patients were recruited for this study, and their baseline characteristics and serum lipid levels are detailed in Table 1. Among them, 112 were male (56%) and 88 were female (44%), with a median age of 61 years (range, 14–83 years). The healthy control group consisted of 185 individuals matched for age and gender with the DLBCL cohort (Supplementary Figure S1A,B). Comparison of lipid levels between the DLBCL group and the healthy control group revealed that DLBCL patients had significantly lower levels of TC, HDL-C, LDL-C, Lp(a), ApoA1, and ApoB compared to healthy controls (p < 0.05), while ApoE levels were significantly higher in DLBCL patients (p < 0.05, Figure 1). These results suggest that significant alterations in serum lipid levels are closely associated with the development and progression of DLBCL.

### 3.2 Serum lipid level in relation to DLBCL staging and IPI score

The Ann Arbor staging system is widely used for staging malignant lymphomas, including Hodgkin lymphoma (HL) and non-Hodgkin lymphoma (NHL), and plays a critical role in guiding treatment decisions and prognostic evaluations. The International Prognostic Index (IPI) score is another essential tool for assessing the prognosis of NHL patients. In this study, we evaluated all patients using the IPI scoring criteria and the Ann Arbor staging system and analyzed the relationship between serum lipid levels and these two parameters. The results revealed that low levels of HDL-C and APOA1 were significantly associated with higher IPI scores (p < 0.05, Supplementary Figure S1C,D). Additionally, HDL-C, APOA1, and TC levels were correlated with Ann Arbor staging, with lower levels corresponding to higher Ann Arbor stages (III-IV) (p < 0.05, Supplementary Figure S1E-G). These findings indicate that HDL-C, APOA1, and TC levels are significantly associated with disease staging in DLBCL and have important prognostic value.

### 3.3 Relationship between serum lipid levels and outcome

**FIGURE 2:**
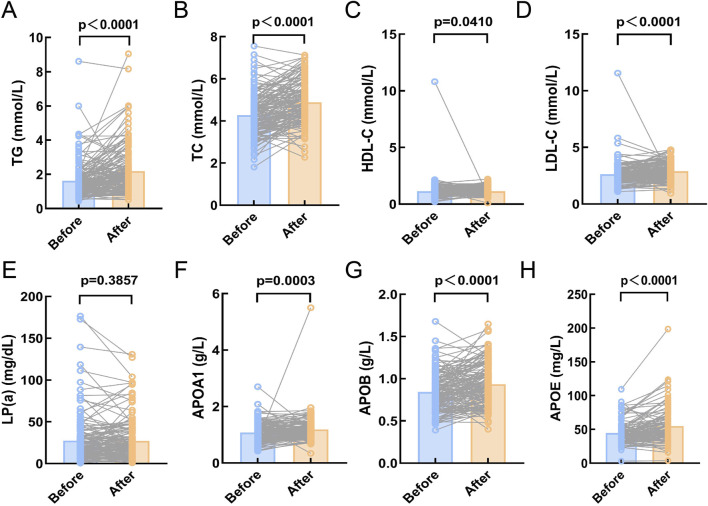
Compare the serum lipid levels of DLBCL patients in CR/PR before and after chemotherapy. **(A-H)**: The expression levels of TG, TC, HDL-C, LDL-C, APOA1, APOB, and APOE were significantly higher after chemotherapy compared to before chemotherapy (p < 0.05). **(E)**: There was no significant difference in the expression level of Lp(a) before and after chemotherapy (p > 0.05). Abbreviations: TG, Triglyceride; TC, Total Cholesterol; HDL-C, High Density Lipoprotein Cholesterol; LDL-C, Low Density Lipoprotein Cholesterol; Lp(a), Lipoprotein A; APOA1, Apolipoprotein A1; APOB, Apolipoprotein B; APOE, Apolipoprotein E.

This study included 200 DLBCL patients who received at least one cycle of chemotherapy, and their treatment outcomes were evaluated following the completion of therapy. Of these, 158 patients achieved complete or partial remission (CR/PR). To further explore the relationship between serum lipid levels at diagnosis and treatment efficacy, paired comparisons of serum lipid levels before and after treatment were conducted in patients who achieved complete or partial remission. The results demonstrated that post-treatment levels of TG, TC, LDL-C, ApoA1, ApoB, and APOE were significantly increased (p < 0.05, Figure 2), while HDL-C and Lp(a) levels showed no significant changes (p > 0.05, Figure 2). Conversely, no significant changes in serum lipid levels were observed in the 14 non-CR/PR patients, which may partly explain their lack of therapeutic efficacy (p > 0.05, Supplementary Figure S2).

### 3.4 Serum lipid levels are significantly associated with prognosis

**FIGURE 3:**
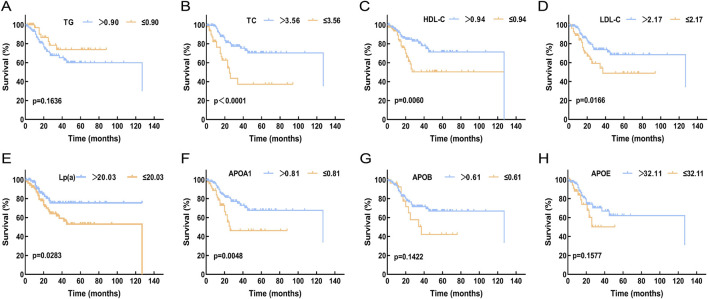
Compare the relationship between the expression levels of serum lipid levels and prognosis in DLBCL patients. **(A)** The expression level of TG showed no significant correlation with OS (p > 0.05). **(B-F)**: High expression levels of TC, HDL-C, LDL-C, Lp(a), and APOA1 were associated with better OS (p < 0.05). **(G,H)**: The expression levels of APOB and APOE showed no significant correlation with OS (p > 0.05). Abbreviations: TG, Triglyceride; TC, Total Cholesterol; HDL-C, High Density Lipoprotein Cholesterol; LDL-C, Low Density Lipoprotein Cholesterol; Lp(a), Lipoprotein A; APOA1, Apolipoprotein A1; APOB, Apolipoprotein B; APOE, Apolipoprotein E.

The relationship between survival outcomes and serum lipid levels was analyzed in 200 DLBCL patients. Optimal cutoff values for overall survival (OS) were determined using the X-tile program for TG, TC, HDL-C, LDL-C, Lp(a), ApoA1, ApoB, and APOE, which were 0.9 mmol/L, 3.56 mmol/L, 0.94 mmol/L, 2.17 mmol/L, 20.03 mg/dL, 0.81 g/L, 0.61 g/L, and 32.11 mg/L, respectively (Supplementary Figure S3). Kaplan-Meier analysis revealed that DLBCL patients with higher levels of TC, HDL-C, LDL-C, Lp(a), and ApoA1 had significantly prolonged OS compared to those with lower lipid levels (p < 0.05, Figure 3).

### 3.5 Univariate and multivariate cox regression analyses of prognostic factors

To identify potential prognostic factors, univariate and multivariate Cox regression analyses were performed for OS (Table 2). Univariate analysis revealed that age, IPI score, ECOG score, Ann Arbor stage, lactate dehydrogenase (LDH), TC, HDL-C, LDL-C, and ApoA1 were significantly associated with OS (p < 0.05). In the multivariate analysis, after excluding known prognostic factors (such as age, IPI score, ECOG score, Ann Arbor stage, and LDH), serum lipid index that were associated with OS in the univariate analysis were incorporated into the Cox proportional hazards regression model. The results demonstrated that TC was an independent prognostic factor for DLBCL (p < 0.05).

**TABLE 2:** Univariate and multivariate Cox hazards analysis for OS in DLBCL.

Variable		Univariate analysis		Multivariate analysis	
	Parameters	HR (95% CI)	p-value	HR (95% CI)	p-value
Male	Male vs. female	0.79 (0.44, 1.44)	0.441		
Age	≥60 vs. < 60	2.67 (1.41, 5.07)	**0.003**		
IPI	>2 vs. ≤ 2	3.04 (1.70, 5.43)	**<0.001**		
ECOG	>1 vs. ≤ 1	2.69 (1.35, 5.37)	**0.005**		
GCB	Yse or no	1.66 (0.94, 2.92)	0.081		
Ann Arbor stage	≥III vs. < III	3.00 (1.49, 6.02)	**0.002**		
LDH (U/L)	>502.49 or ≤502.49	3.29 (1.61, 6.75)	**0.001**		
Statins	“yes” vs. “no”	0.625 (0.26, 1.48)	0.286		
Diabetes	“yes” vs. “no”	0.560 (0.27, 1.16)	0.118		
TG (mmol/L)	>0.90 or ≤0.90	1.80 (0.80, 4.02)	0.154		
TC (mmol/L)	>3.56 or ≤3.56	0.30 (0.17, 0.53)	**<0.001**	0.40 (0.17, 0.96)	**0.041**
HDL-C (mmol/L)	>0.94 or ≤0.94	0.44 (0.25, 0.78)	**0.005**	0.68 (0.32, 1.46)	0.328
LDL-C (mmol/L)	>2.17 or ≤2.17	0.47 (0.26, 0.84)	**0.011**	0.92 (0.41, 2.07)	0.833
Lp (a) (mg/dL)	>20.03 or ≤20.03	0.55 (0.30, 1.00)	0.050		
APOA1 (g/L)	>0.81 or ≤0.81	0.42 (0.23, 0.75)	**0.003**	0.74 (0.34, 1.63)	0.460
APOB (g/L)	>0.61 or ≤0.61	0.61 (0.31, 1.24)	0.172		
APOE (mg/L)	>32.11 or ≤32.11	0.62 (0.30, 1.29)	0.202		

Bold type indicates statistical significance (p < 0.05).

### 3.6 The role of cholesterol metabolism in DLBCL

**FIGURE 4:**
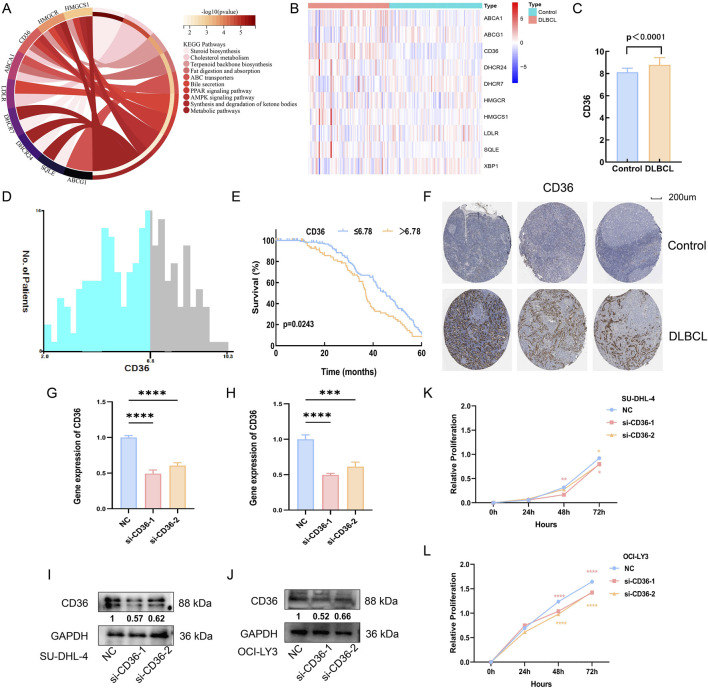
The role of cholesterol metabolism in DLBCL. **(A)** KEGG enrichment analysis of cholesterol metabolism-related genes; **(B)** Heatmap analysis of the differential expression of cholesterol metabolism genes in the blood of healthy controls and DLBCL patients; **(C)** CD36 expression is higher in DLBCL patients compared to healthy controls (p < 0.05); **(D,E)**: X-tile analysis identifies 6.78 as the optimal cutoff value for CD36, and patients with high CD36 expression in DLBCL have a poorer prognosis (p < 0.05); **(F)** Immunohistochemistry indicates that CD36 expression is higher in non-Hodgkin lymphoma compared to normal lymph nodes (p < 0.05). **(G,H)**: CD36 mRNA expression levels in SU-DHL-4 and OCI-LY3 cells, respectively, after transfection with CD36-specific siRNA. **(I,J)**: CD36 protein expression levels in SU-DHL-4 and OCI-LY3 cells, respectively, following transfection with CD36-targeting siRNA. **(K,L)**: Proliferation levels of SU-DHL-4 and OCI-LY3 cells, respectively, after transfection with CD36 siRNA.

KEGG analysis revealed that these genes are closely associated with cholesterol metabolism (Figure 4A). We analyzed the differential expression of these genes between healthy controls and DLBCL patients using blood sample data from 87 healthy controls and 76 DLBCL patients sourced from the GEO database. It is evident that the expression of CD36, a key mediator of *de novo* cholesterol synthesis, is significantly elevated in DLBCL compared to healthy controls (Figures 4B,C). Additionally, we extracted sequencing and survival data from 223 DLBCL patients in the GEO database. The optimal cutoff value for overall survival (OS) was determined using the X-tile program (Figure 4D). Our analysis demonstrated that high expression of CD36, a key mediator of cholesterol synthesis, was significantly associated with poor prognosis (p < 0.05, Figure 4E). Furthermore, immunohistochemical analysis from the ProteinAtlas database confirmed that CD36 expression was markedly elevated in NHL compared to normal lymph nodes (p < 0.05, Figure 4F). Collectively, these findings suggest that cholesterol metabolism may play a crucial role in promoting tumor growth in DLBCL.

Next, to further elucidate the functional role of CD36 in DLBCL cell proliferation, we transfected SU-DHL-4 and OCI-LY3 cell lines with CD36-specific siRNA. Efficient knockdown was confirmed at both the mRNA and protein levels via RT-qPCR and Western blot analysis, respectively (p < 0.05; Figures 4G–J). Subsequent CCK-8 assays revealed that CD36 knockdown significantly impaired the proliferative capacity of DLBCL cells (p < 0.05; Figures 4K,L). These results indicate that CD36 may facilitate tumor growth in DLBCL through modulation of cholesterol metabolism pathways, offering experimental support for its potential utility as a therapeutic target.

### 3.7 Effects of Methy-β-cyclodextrin on proliferation of DLBCL cells

**FIGURE 5:**
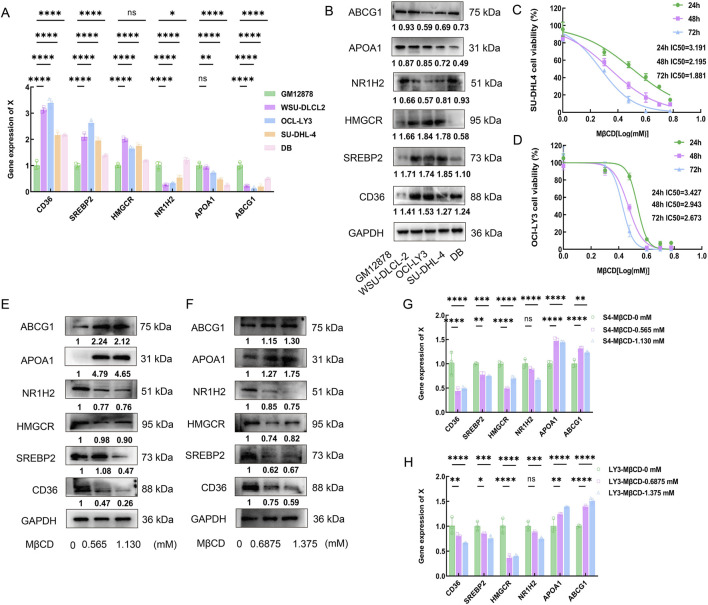
Effects of Methy-β-cyclodextrin on proliferation of DLBCL cells. **(A,B)**: RT-PCR and Western blot (WB) results show the expression levels of CD36, SREBP2, HMGCR, NR1H2, APOA1 and ABCG1 in the normal lymphocyte cell line GM12878 and DLBCL cell lines (WSU-DLCL-2, OCI-LY3, SU-DHL-4, and DB). **(C,D)**: CCK-8 assay was used to evaluate the time- and dose-dependent effects of MβCD on SU-DHL-4 and OCI-LY3 cell lines, and the IC50 values were calculated. SU-DHL-4 and OCI-LY3 cell lines were exposed to MβCD (0, 1, 2, 3, 4, 5, and 6 mmol/L) for 24, 48, and 72 h. Cell viability was measured using the CCK-8 assay, and the half-maximal inhibitory concentration (IC50) values were determined. **(E-H)**: SU-DHL-4 and OCI-LY3 cell lines were treated with Methyl-β-cyclodextrin for 48 h. Western blot and RT-PCR results demonstrated that the expression levels of CD36, SREBP2 and HMGCR decreased in both cell lines, while the expression levels of APOA1 and ABCG1 increased. MβCD:Methyl-β-cyclodextrin.

To further investigate the role of cholesterol metabolism in DLBCL, the expression levels of cholesterol metabolism-related proteins were examined in a normal lymphocyte cell line (GM12878) and DLBCL cell lines (WSU-DLBCL-2, OCI-LY3, SU-DCL-4, and DB). RT-PCR and Western blot (WB) results showed that the expression of cholesterol synthesis-related proteins CD36, SREBP2 and HMGCR was significantly higher in WSU-DLBCL-2, OCI-LY3, and SU-DCL-4 cells compared to GM12878, while the expression of cholesterol efflux-related proteins NR1H2, APOA1 and ABCG1 was significantly lower (Figures 5A,B). These results validated the phenomenon of increased cholesterol synthesis and reduced efflux in DLBCL cells, confirming cholesterol metabolic reprogramming in DLBCL at the cellular level. Furthermore, the effects of the cholesterol inhibitor Methyl-β-cyclodextrin (MβCD) on DLBCL cell growth were explored. The impact of MβCD at different doses and time points on SU-DCL-4 and OCI-LY3 cells was assessed, and the proliferation of both cell lines was significantly inhibited in a dose- and time-dependent manner (Figures 5C,D).

The half-maximal inhibitory concentration (IC50) of MβCD was calculated at different time points through triplicate experiments, and concentrations equivalent to 1/4 and 1/2 of the average IC50 value at 48 h were selected to further validate the effects of MβCD on cholesterol synthesis- and efflux-related proteins in DLBCL cells. The experimental results showed that, after MβCD treatment, the expression levels of CD36, SREBP2 and HMGCR were significantly reduced, whereas the expression levels of APOA1 and ABCG1 were significantly increased (Figures 5E–H). These findings suggest that MβCD may effectively inhibit DLBCL cell growth through a dual mechanism of suppressing cholesterol synthesis and promoting cholesterol efflux.

## 4 Discussion

At the clinical level, a retrospective analysis of 200 DLBCL patients and 185 healthy controls from the Second Affiliated Hospital of Nanchang University revealed significant differences in serum lipid levels between DLBCL patients and healthy controls. DLBCL patients exhibited significantly lower levels of TC, HDL-C, LDL-C, Lp(a), APOA1, and ApoB compared to healthy controls (p < 0.05), while ApoE levels were significantly higher in DLBCL patients (p < 0.05). Among these, HDL-C, APOA1, and TC levels were significantly associated with disease staging in DLBCL patients and held important prognostic value. Patients with higher levels of TC, HDL-C, LDL-C, Lp(a), and APOA1 had better prognoses (p < 0.05). Univariate and multivariate Cox regression analyses demonstrated that pre-treatment TC levels were an independent prognostic factor for OS, providing a new direction for stratified treatment of DLBCL. It is noteworthy that this study also evaluated potential confounders known to influence lipid metabolism, such as a history of diabetes and statin use. Univariate analysis indicated that neither factor was a significant predictor for OS in this cohort (p > 0.05). We speculate that this result may be related to the relatively small sample size and somewhat limited statistical power, which may have hindered the detection of potential subtle effects. Future studies with expanded cohorts are warranted to further elucidate the potential impact of diabetes and statin medication on the survival outcomes of DLBCL patients.

Bioinformatics analysis suggested that these alterations in lipid levels were closely related to cholesterol metabolism and often accompanied by high expression of CD36 and low expression of APOA1. At the mechanistic level, cell experiments confirmed that the expression of proteins and enzymes promoting cholesterol absorption and synthesis (CD36, SREBP2 and HMGCR) was elevated in DLBCL cell lines, while the expression of proteins promoting cholesterol efflux (NR1H2, APOA1 and ABCG1) was reduced, collectively driving the proliferation of DLBCL tumor cells. Following intervention with the cholesterol inhibitor MβCD, the expression of cholesterol synthesis-related proteins (CD36, SREBP2 and HMGCR) decreased, while the expression of cholesterol efflux-related proteins (APOA1 and ABCG1) increased, leading to significant inhibition of tumor cell growth. These results indicate that cholesterol and its metabolites are potential therapeutic targets for DLBCL.

The relationship between serum cholesterol levels and prognosis is complex and heterogeneous across different cancer types. A large meta-analysis involving 24,655 cancer patients demonstrated that low levels of TC and HDL-C were associated with poorer OS, suggesting that TC and HDL-C may act as protective factors in cancer patients (Zhou et al., 2018). However, in breast cancer, elevated TC levels have been linked to an adverse prognosis, potentially due to the increased risk of metastasis associated with high cholesterol levels (Nelson, 2018). Previous clinical studies on DLBCL identified TC and HDL-C as favorable prognostic factors (Wang et al., 2023; Gao et al., 2018), a finding consistent with the results of this study. Nevertheless, the specific regulatory mechanisms of cholesterol metabolism in DLBCL remain unclear, warranting further in-depth investigation.

Lipid metabolism undergoes significant reprogramming in cancer, with cholesterol metabolic reprogramming being one of the most common forms of lipid reprogramming (Faubert et al., 2020; Cheng et al., 2018). The processes of cholesterol synthesis, absorption, storage, and efflux play crucial roles in tumor growth (Xu et al., 2020). As a key component of cell membranes, cholesterol is a fundamental building block of the phospholipid bilayer, primarily maintaining membrane stability and regulating its fluidity (de Boer et al., 2018). During tumor development, cholesterol is essential for cell proliferation and membrane biosynthesis (Silvente-Poirot and Poirot, 2012). Cells acquire cholesterol through two primary pathways: The first involves receptor-mediated endocytosis of low-density lipoprotein (LDL) particles via the LDL receptor (LDL-R), facilitating cholesterol uptake from the blood into cells (Bilot et al., 2020). In addition, the transmembrane glycoprotein CD36, which acts as a receptor for fatty acids and cholesteryl esters, enhances cellular cholesterol uptake by directly binding to LDL particles and promoting their adsorption and internalization at the cell surface (Ulug and Nergiz-Unal, 2021). This mechanism partially accounts for the observed decrease in serum cholesterol levels and concomitant increase in intracellular cholesterol content in patients with diffuse large B-cell lymphoma (DLBCL). The second pathway involves the transport of the precursor acetyl-CoA into cells by CD36, where *de novo* cholesterol synthesis occurs through biochemical reactions catalyzed by rate-limiting enzymes such as 3-hydroxy-3-methylglutaryl-CoA reductase (HMGCR) and squalene monooxygenase (SQLE), along with the master regulator of cholesterol synthesis, SREBP2 (Steck and Lange, 2010).

Studies have shown that CD36 is upregulated in various cancers, including acute myeloid leukemia, breast cancer, colorectal cancer, and gastric cancer. Both *in vitro* and *in vivo* experiments have confirmed that CD36 regulates tumor growth, metastasis, and drug resistance through multiple molecular mechanisms (Wang et al., 2020; Farge et al., 2023). In this study, CD36 expression was significantly elevated and associated with poor prognosis in DLBCL. Functionally, siRNA-mediated knockdown of CD36 markedly suppressed the proliferation of DLBCL cells, underscoring its crucial role in promoting tumor growth. Compared to normal lymphocyte cell lines, the expression of key regulatory factors for *de novo* cholesterol synthesis, such as SREBP2 and the rate-limiting enzyme HMGCR, was significantly higher in DLBCL cell lines. This suggests that CD36 may drive cholesterol metabolic reprogramming in DLBCL by regulating *de novo* cholesterol synthesis in tumor cells, altering their metabolic patterns and acting as a critical driver of cholesterol metabolism in DLBCL.

The efflux of intracellular cholesterol primarily relies on receptor-mediated transport proteins that facilitate the movement of cholesterol from inside the cell to the extracellular space or cell membrane (Juhl et al., 2022). Intracellular cholesterol is transported to the extracellular space by ABCA1 and ABCG1, where it binds to HDL to form mature HDL particles, participating in reverse cholesterol transportIntracellular cholesterol is transported to the extracellular space by ABCA1 and ABCG1, where it binds to HDL to form mature HDL particles, participating in reverse cholesterol transport (Yu and Tang, 2019). NR1H2 (also known as LXRβ, liver X receptor beta) promotes cholesterol efflux by activating the expression of ATP-binding cassette transporters, such as ABCA1 and ABCG1 (Nelson et al., 2017). Studies have shown that these genes regulating cholesterol efflux play important roles in the progression of various cancers by modulating lipid metabolism, inflammation, and immune evasion (Savic et al., 2016; Xinyi and Gong, 2024). In this study, NR1H2 and ABCG1 expression levels were significantly higher in DLBCL compared to healthy controls, consistent with the aforementioned findings. This further supports the potential role of cholesterol efflux mechanisms in DLBCL.

Additionally, APOA1 is involved in intracellular cholesterol efflux. As the primary protein component of HDL-C, APOA1 binds to ATP-binding cassette (ABC) transporters, accepting excess cholesterol from cells and facilitating its reverse transport to the liver for metabolism, thereby maintaining cholesterol homeostasis (Luo et al., 2020). Moreover, APOA1 possesses anti-inflammatory properties, modulates immune responses, and inhibits tumor cell proliferation and metastasis by reducing intracellular cholesterol levels (Georgila et al., 2019). Although serum APOA1 levels were not identified as independent prognostic factors for OS in the clinical analysis of this study, low expression was significantly associated with poor prognosis. Additionally, the statistically significant differences in APOA1 levels before and after treatment in patients achieving CR and PR confirmed that downregulation of APOA1 expression promotes DLBCL progression by reducing cholesterol efflux in tumor cells.

The limitations of this study include: First, the clinical sample size is relatively small, requiring further expansion to validate the predictive value of serum cholesterol as an independent prognostic marker for DLBCL. Second, the absence of *in vivo* models (e.g., mouse xenografts) to verify the efficacy and toxicity of MβCD highlights the need for further exploration of its clinical translational potential.

In summary, this study confirms cholesterol metabolic reprogramming in DLBCL, suggesting that targeting cholesterol and its metabolites may represent a novel therapeutic strategy. However, its clinical application requires further validation through multicenter, prospective studies. Current research primarily focuses on the correlation between serum lipid levels and DLBCL prognosis, while in-depth exploration of cholesterol metabolism mechanisms remains limited. Future studies should focus on elucidating the specific mechanisms of cholesterol and its related metabolites in DLBCL and developing targeted therapies to improve patient outcomes. Additionally, the reliability and utility of serum cholesterol levels as prognostic indicators must be validated through broader clinical research.

## Data Availability

The original contributions presented in the study are included in the article/Supplementary Material, further inquiries can be directed to the corresponding author.
