# Supplementary material for: Cholesterol metabolic reprogramming drives the onset of DLBCL and represents a promising therapeutic target
Source: Front Cell Dev Biol. 2025 Sep 17;13:1585521. doi: 10.3389/fcell.2025.1585521 (PMC12484024; doi:10.3389/fcell.2025.1585521)
Supplement: Supplementary file 1 [file Table1.docx]

### ****Supplementary Table 1. List of Antibodies Used in the Study****

| **Target Antigen** | **Catalog Number** | **Company (Country)** |
| --- | --- | --- |
| ****Primary Antibodies**** |  |  |
| NR1H2 | A04523-2 | Boster, China |
| HMGCR | A00643-3 | Boster, China |
| GAPDH | HRP-60004 | Proteintech Group, China |
| CD36 | 66395-1-lg | Proteintech Group, China |
| SREBP2 | 28212-1-AP | Proteintech Group, China |
| ABCG1 | 13578-1-AP | Proteintech Group, China |
| APOA1 | 14427-1-AP | Proteintech Group, China |
| ****Secondary Antibodies**** |  |  |
| Anti-Rabbit IgG (HRP) | SA00001-2 | Proteintech Group, China |
| Anti-Mouse IgG (HRP) | SA00001-1 | Proteintech Group, China |
