# Supplementary material for: Cholesterol metabolic reprogramming drives the onset of DLBCL and represents a promising therapeutic target
Source: Front Cell Dev Biol. 2025 Sep 17;13:1585521. doi: 10.3389/fcell.2025.1585521 (PMC12484024; doi:10.3389/fcell.2025.1585521)
Supplement: Supplementary file 2 [file DataSheet1.docx]

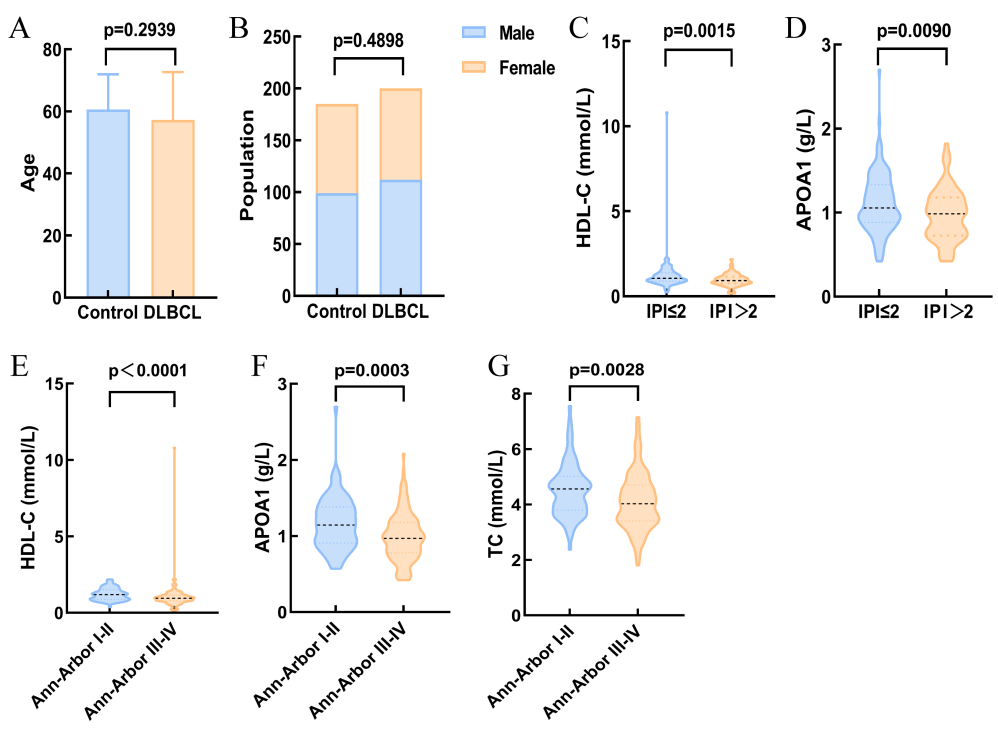


Figure. S1 The relationship between serum lipid levels and IPI score as well as Ann-Arbor staging in DLBCL. A-B: Differential analysis confirmed that there were no significant differences in gender and age between the Control group and the DLBCL group, indicating comparability (p＞0.05). C-D: After dividing DLBCL patients into two groups based on IPI score (IPI ≤ 2 and IPI > 2), serum lipid levels were compared between the two groups. The results showed that HDL-C and APOA1 levels were higher in the IPI ≤ 2 group (p＜0.05). E-F: After dividing DLBCL patients into two groups based on Ann-Arbor staging (Ann-Arbor I-II and Ann-Arbor III-IV), serum lipid levels were compared between the two groups. The results indicated that HDL-C, APOA1, and TC levels were higher in the Ann-Arbor I-II group (p＜0.05). Abbreviations: TG, Triglyceride; TC, Total Cholesterol; HDL-C, High Density Lipoprotein Cholesterol; LDL-C, Low Density Lipoprotein Cholesterol; Lp(a), Lipoprotein A; APOA1, Apolipoprotein A1; APOB, Apolipoprotein B; APOE, Apolipoprotein E.


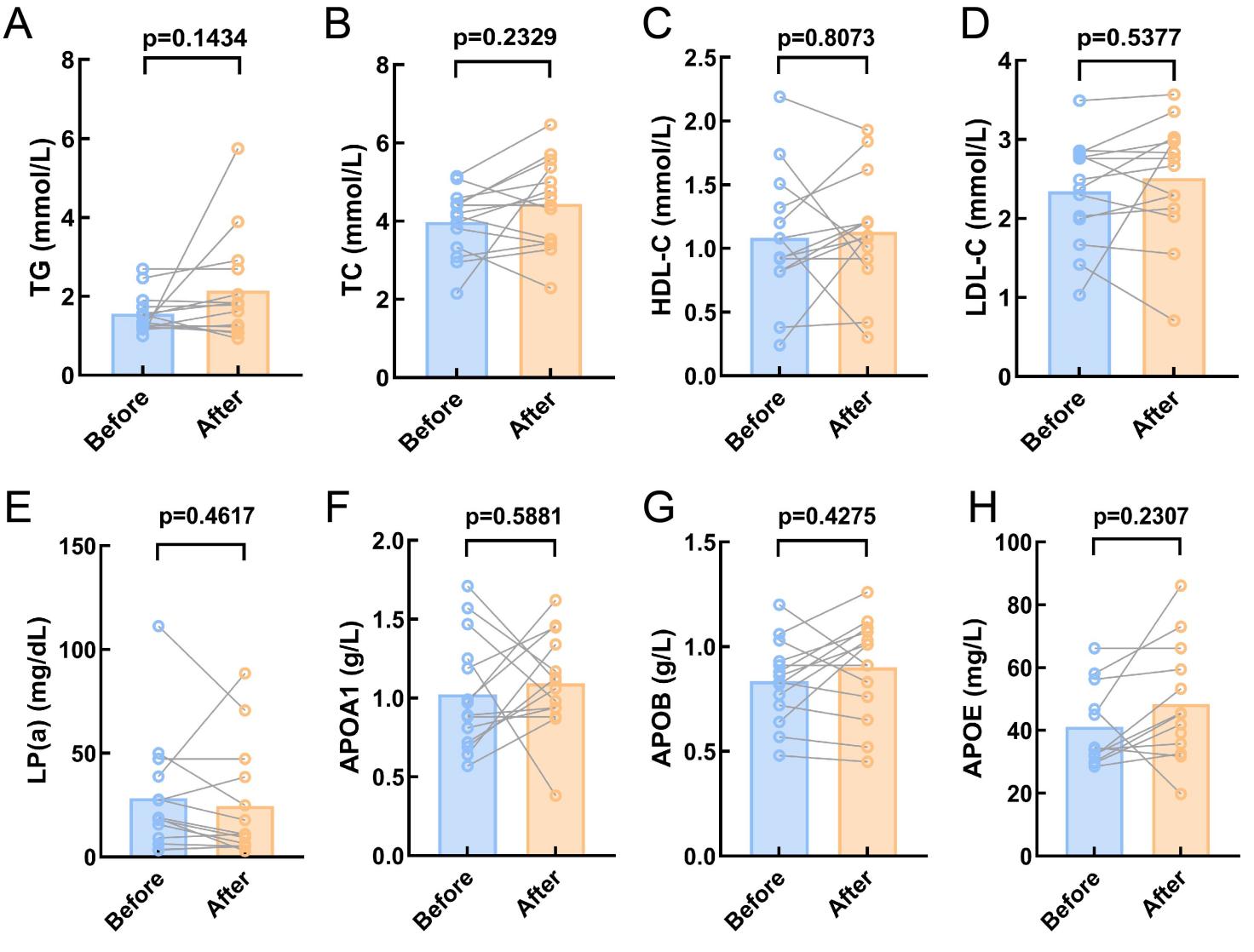


Figure. S2 Compare the serum lipid levels of DLBCL patients in non-CR/PR before and after chemotherapy. A-H: No significant differences were observed in serum lipid levels before and after treatment in DLBCL patients who achieved non-CR/PR (p > 0.05). Abbreviations: TG, Triglyceride; TC, Total Cholesterol; HDL-C, High Density Lipoprotein Cholesterol; LDL-C, Low Density Lipoprotein Cholesterol; Lp(a), Lipoprotein A; APOA1, Apolipoprotein A1; APOB, Apolipoprotein B; APOE, Apolipoprotein E.


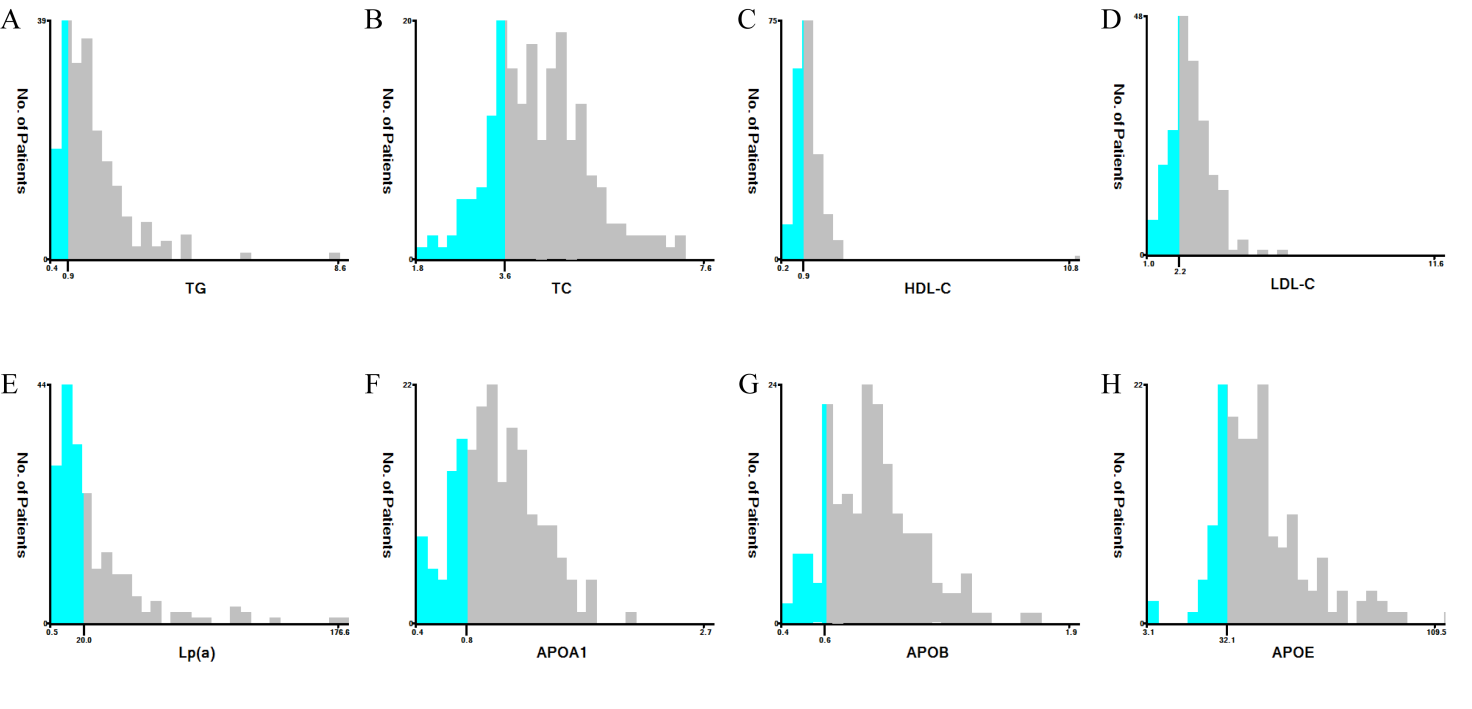


Figure S3 The optimal cutoff values for serum lipid levels in overall survival (OS) were determined using the X-tile program. A: TG (triglycerides) at 0.9 mmol/L; B: TC (total cholesterol) at 3.56 mmol/L; C: HDL-C (high-density lipoprotein cholesterol) at 0.94 mmol/L; D: LDL-C (low-density lipoprotein cholesterol) at 2.17 mmol/L; E: Lp(a) (lipoprotein(a)) at 20.03 mg/dL; F: APOA1 (apolipoprotein A1) at 0.81 g/L; G: APOB (apolipoprotein B) at 0.61 g/L; H: APOE (apolipoprotein E) at 32.11 mg/L. Abbreviations: TG, Triglyceride; TC, Total Cholesterol; HDL-C, High Density Lipoprotein Cholesterol; LDL-C, Low Density Lipoprotein Cholesterol; Lp(a), Lipoprotein A; APOA1, Apolipoprotein A1; APOB, Apolipoprotein B; APOE, Apolipoprotein E.
